# Genetic testing results in Slovenian male breast cancer cohort indicate the BRCA2 7806-2A > G founder variant could be associated with higher male breast cancer risk

**DOI:** 10.1007/s10549-021-06224-5

**Published:** 2021-04-23

**Authors:** Ksenija Strojnik, Mateja Krajc, Vita Setrajcic Dragos, Vida Stegel, Srdjan Novakovic, Ana Blatnik

**Affiliations:** 1 Institute of Oncology, Cancer Genetics Clinic, Ljubljana, Slovenia; 2 University of Ljubljana, Ljubljana, Slovenia; 3 Department of Molecular Diagnostics, Institute of Oncology, Ljubljana, Slovenia

**Keywords:** Male breast cancer, *BRCA1*, *BRCA2*, Hereditary breast cancer, Founder variant

## Abstract

**Purpose:**

To analyze the prevalence of pathogenic/likely pathogenic variants (P/LPVs) in *BRCA1* and *BRCA2* genes in the largest cohort of Slovenian male breast cancer (MBC) patients to date and to explore a possible correlation between the Slovenian founder variant *BRCA2*:c.7806-2A > G and predisposition to MBC.

**Methods:**

We performed a retrospective analysis of 81 MBC cases who underwent genetic counseling and/or testing between January 1999 and May 2020. To explore a possible genotype–phenotype correlation, we performed additional analyses of 203 unrelated families with P/LPVs in *BRCA2* and 177 cases of female breast cancer (FBC) in carriers of P/LPVs in *BRCA2*.

**Results:**

Detection rate of P/LPVs in the *BRCA1* and *BRCA2* genes was 24.7% (20/81) with 95% of them in *BRCA2* gene. The only two recurrent P/LPVs were *BRCA2*:c.7806-2A > G and *BRCA2*:c.3975_3978dupTGCT (9 and 5 MBC cases, respectively). In families with *BRCA2*:c.7806-2A > G, the incidence of MBC cases was higher compared to families with other P/LPVs in *BRCA2*; however, the difference did not reach statistical significance (17.8% vs. 8.9%, *p* = 0.105). *BRCA2*:c.7806-2A > G was detected in both families with multiple cases of MBC. This splice-site variant represented a significantly higher proportion of all *BRCA2* P/LPVs detected in MBC carriers compared to FBC carriers (47.4% vs. 26%, *p* = 0.049).

**Conclusion:**

We observed a high mutation detection rate and conclude this may be due to the prevalent *BRCA2*:c.7806-2A > G variant in Slovenia. Our results indicate a possible association between this variant and higher risk of breast cancer in males compared to other identified P/LPVs in *BRCA2*.

## Introduction

Male breast cancer (MBC) is a rare disease, comprising up to 1% of all breast cancers and up to 1% of all cancers in males [1–3]. Age-adjusted incidence rate in Slovenia is reported to be 1.1 per 100,000 and is in line with incidence rates reported in other Caucasian populations [2, 4, 5]. Various demographic, environmental, hormonal, and genetic factors have been associated with MBC [2, 6]. Among genetic factors, mutations in *BRCA1* and, especially, *BRCA2* genes are the most clearly established predispositions [2]. Genetic testing is recommended for all MBC patients in order to guide screening recommendations for other types of cancer and to identify other family members at risk [1, 7], as well as for treatment with PARP (poly-ADP ribose polymerase) inhibitors in metastatic settings according to the American Society of Clinical Oncology 2020 guidelines [8].

In 2008, Besic together with members of our group published first results of genetic testing in a small cohort of 25 Slovenian MBC patients and reported a high prevalence of 16% of pathogenic/likely pathogenic variants (P/LPVs) in *BRCA2* [9]. In three out of four MBC carriers of P/LPVs in *BRCA2*, the highly recurrent Slovenian founder splice-site variant *BRCA2*:c.7806-2A > G (formerly known as IVS16-2A > G) was detected [10, 11]. A group from Aviano, Italy, also reported this variant as recurrent in northeast part of Italy and suggested an association between this splice-site *BRCA2* variant and risk of breast cancer in males [12–14].

The aim of our study was to analyze the prevalence of P/LPVs in *BRCA1* and *BRCA2* genes in the largest cohort of Slovenian MBC patients to date. We performed a retrospective analysis of all MBC cases who were referred to our Cancer Genetic Clinic at the Institute of Oncology Ljubljana, Slovenia, since its inception in 1999. Our primary objective was to re-evaluate the high detection rate of P/LPVs in *BRCA1* and *BRCA2* genes in a small cohort of Slovenian MBC patients, reported previously by Besic et al. in 2008 [9]. In addition, we wanted to document the spectrum of all P/LPVs as well as possible difference between carriers and non-carriers of P/LPV in *BRCA1* and *BRCA2* genes. Our secondary objective was to explore a possible correlation between the *BRCA2*:c.7806-2A > G variant and predisposition to MBC compared to other *BRCA2* P/LPVs identified in our cohort. We also evaluated certain clinical characteristics of MBC in carriers of *BRCA2*:c.7806-2A > G compared to other P/LPVs in *BRCA2*.

## Methods

### MBC cohort and data collection

We performed a retrospective analysis of MBC cases identified in the Cancer Genetics Clinic’s database at the Institute of Oncology Ljubljana, the only comprehensive cancer center in Slovenia. Our cohort is unselected for family history of cancer, since MBC has always been one of the independent inclusion criteria for genetic testing at the Institute [11]. All MBC cases referred to our Clinic since the introduction of genetic counseling and testing in 1999 up to May 2020 who opted for genetic testing were included. Only P/LPVs in *BRCA1* and *BRCA2* genes were analyzed.

For the purpose of this study, positive family history for hereditary breast and ovarian cancer syndrome (HBOC-related) cancers was defined as a family history of female breast, ovarian, prostate, and/or pancreatic cancer in first and/or second-degree relatives. As part of our routine clinical protocol, all probands are required to report their family history of cancer, which is then verified in the Cancer Registry of Republic of Slovenia, a national system of mandatory reported cancer cases, established in 1950. Also, we used this database to check if the MBC patients in our cohort had been diagnosed with any other cancers.

Information on relevant MBC clinical and pathological characteristics was retrieved from the Institute’s electronic database and patients’ medical records. We applied surrogate definitions of intrinsic subtypes of breast cancer as defined by the St. Galen International Expert Consensus 2013 [15].

For the purpose of exploring possible genotype–phenotype correlation between the *BRCA2*:c.7806-2A > G variant and predisposition to MBC compared to other *BRCA2* P/LPVs identified at our Institute, we performed an additional analysis of all unrelated families with P/LPVs in *BRCA2* detected at our Institute since 1999. We recorded all cases of MBC in these families. Also, all cases of breast cancer in female carriers of P/LPVs in *BRCA2* were identified from the Cancer Genetics Clinic’s database.

We obtained the approval for our analysis from the Institute’s Committee for Medical Ethics (#ERIDEK-0069/2020).

### Genetic testing methods

**Fig. 1:**
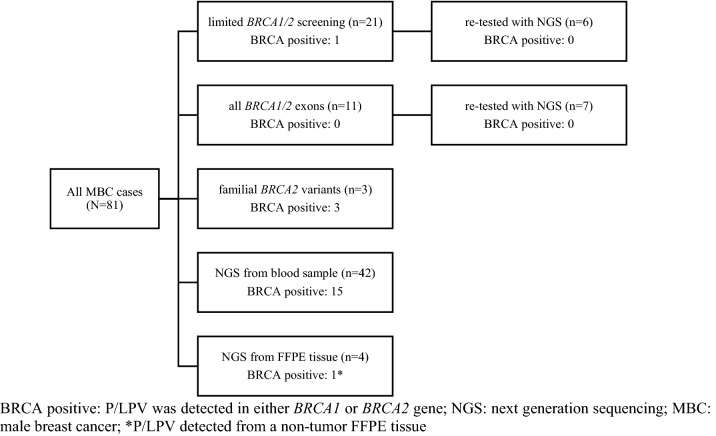
Flow chart of genetic testing methods used and detection of P/LPVs in *BRCA1* and *BRCA2*. BRCA positive: P/LPV was detected in either *BRCA1* or *BRCA2* gene; NGS: next-generation sequencing; MBC: male breast cancer; *P/LPV detected from a non-tumor FFPE tissue

Genetic testing was performed at the Department of Molecular Diagnostics at the Institute as well as other laboratories [11]. Together, we identified 81 MBC cases. Different genetic testing methods were used. In 55 MBC cases, genetic testing with next-generation sequencing (NGS) was performed on blood samples. Of these, 54 were tested using NGS of targeted panels Illumina’s TruSight Cancer Panel or TruSight Hereditary Cancer Panel [16]. Large intragenic deletions in *BRCA1* and *BRCA2* genes were detected from NGS data with copy number analysis using SeqNext v4.4.0 (JSI medical systems) or with multiplex ligation-dependent probe amplification (MLPA). Identified P/LPVs in *BRCA1* and *BRCA2* were confirmed using Sanger sequencing or MLPA analysis from separate blood samples. In one patient, clinical exome sequencing was performed at another laboratory as previously described by Bergant et al. [17]. In 15 MBC cases, who were tested prior to the introduction of NGS in 2014, only a limited mutational screen for the six highly recurrent P/LPV in *BRCA1* and *BRCA2* in Slovenian population (c.7806-2A > G in *BRCA2*; c.5266dupC, c.1687C > T. c.191G > A, c.181 T > G and c.181 T > A in *BRCA1*) was performed. In these, denaturing gradient gel electrophoresis (DDGE) for exon 4, part of exon 10 and exon 19 of BRCA1, DDGE for exon 17 of *BRCA2*, and in two cases additionally protein truncation test (PTT) for exon 10 of *BRCA1* and exon 11 of *BRCA2* were used as detailed elsewhere by our group [11]. In four MBC cases, complete screening of all *BRCA1* and *BRCA2* exons with combination of high-resolution melting (HRM), DDGE, and Sanger sequencing methods as well as MLPA for detecting large genomic deletions was performed as previously described [18, 19]. Out of 55 patients who underwent NGS testing, 42 were previously untested and 13 had already tested negative with previously performed genetic screening using DGGE, HRM, PPT, or Sanger sequencing; all living MBC patients who tested negative prior to the introduction NGS at our Institute in 2014 were re-contacted, and 13 of them responded and opted for re-testing with NGS. In three MBC cases, only Sanger sequencing was performed to determine known familial pathogenic variants in *BRCA2* gene. In four MBC cases, NGS testing (TruSight Tumor 170) was performed on archived formalin-fixed paraffin-embedded (FFPE) tissue samples (two samples were obtained from tumor tissue and two from non-tumor) as previously described by Klancar et al. [20]. Flow chart of genetic testing methods performed as well as detection of P/LPVs in *BRCA1* and *BRCA2* with different testing methods used is depicted in the Fig. 1.

### Statistical analysis

We used descriptive statistics to describe relevant MBC patients' clinical, pathological, and genetic as well as their families’ characteristics. Association between categorical variables was evaluated using Pearson’s chi-squared or Fisher's exact test, as appropriate. To compare means, we used the independent-samples t-test. All significance tests were two-sided using an alpha level of 0.05. The statistical analysis was performed with the licensed IBM SPSS Statistics software, version 25.

## Results

### MBC cohort characteristics

We identified 81 MBC cases from 80 unrelated families who underwent genetic counseling and/or testing at our Institute between January 1999 and May 2020. Pathological and clinical characteristics of MBC cases are summarized in Table 1. Patients were diagnosed with breast cancer between 1970 and 2019 with half of them diagnosed prior to 2011. Excluding non-melanoma skin cancer and contralateral breast cancer, 19/81 (23.5%) MBC patients had a personal history of additional malignant primary tumors. Melanoma was reported in four MBC patients, colorectal cancer in three, prostate cancer in three, renal cell carcinoma in two, hematological malignancy in two, lung cancer in one, conjunctival squamous cell carcinoma in one, unknown primary in one, while two patients had multiple additional primary tumors (prostate and gastric cancer; prostate and lung cancer). In 31/80 (38.7%) of unrelated families with MBC cases, there was a family history of female breast, ovarian, prostate, and/or pancreatic cancer, and in 2/80 (2.5%), there was a family history of male breast cancer (a brother in one and a father in the other).

**Table 1:** Characteristics of 81 male breast cancer cases

Characteristic	N (%)
Age (median)	
At diagnosis	62 (range 17–87)
At genetic testing	66 (range 39–88)
MBC histology	
DCIS	4 (4.9)
IDC	70 (86.4)
ILC	1 (1.2)
Other^a^	6 (7.5)
Stage	
0	4 (4.9)
I-III	72 (88.9)
IV	5 (6.2)
Intrinsic subtypes (*n* = 55)^b^	
Luminal A	20 (36.4)
Luminal B	26 (47.3)
Luminal B HER2 +	7 (12.7)
HER2 +	1 (1.8)
Basal	1 (1.8)
Contralateral MBC	
Yes	2 (2.5)
No	79 (97.5)
Relapse	
Yes	17 (21)
No	59 (72.8)
Primarily metastatic	5 (6.2)

*DCIS* ductal carcinoma in situ, *IDC* invasive ductal carcinoma, *ILC* invasive lobular carcinoma

^a^Mixed IDC and ILC (1), secretory (2), mucinous (1), encapsulated papillary with invasion (1), only cytology available (1)

^b^Data not available for 22 invasive MBC cases

### Detection rate and spectrum of P/LPVs in *BRCA1* and *BRCA2*

Detection rate of P/LPVs in the *BRCA1* and *BRCA2* genes was 24.7% (20/81), with 19/81 (23.5%) detected in *BRCA2* and 1/81 (1.2%) in *BRCA1*. Among all, 95% (19/20) of identified P/LPVs were in *BRCA2* gene. Spectrum of detected P/LPVs together with clinical and pathological characteristics of MBC cases, and personal and family history of additional cancers are detailed in Table 2. Detection rate in MBC cases with a family history of other HBOC-related cancers in first- and/or second-degree relatives was significantly higher compared to those without (43.7% (14/32) vs. 12.2% (6/49), *p* = 0.001).

**Table 2:** Spectrum of detected P/LPVs in *BRCA1* and *BRCA2*

MBC No	Gene	HGVS nomenclature		MBC characteristics: histology, intrinsic subtype (age at diagnosis)	Other cancers (age at diagnosis)	Family history of HBOC-related cancers (age at diagnosis)
		Nucleotide change	Protein change			
1	*BRCA1*	e4-9del	p.?	IDC, luminal A (48)	–	–
2	*BRCA2*	c.7806-2A > G	p.?	IDC, luminal B (64)	–	MBC (64); 4 FBC (30,61,70,79); bil FBC (68)
3	*BRCA2*	c.7806-2A > G	p.?	DCIS (56)	–	–
4	*BRCA2*	c.7806-2A > G	p.?	IDC, luminal B HER2 + (64)	–	MBC (64); 4 FBC (30,61,70,79); bil FBC (68)
5	*BRCA2*	c.7806-2A > G	p.?	IDC, luminal B (58)	M (68)	MBC (72); bil FBC (43,50); FBC (62)
6	*BRCA2*	c.7806-2A > G	p.?	IDC, luminal B (38)	–	FBC (65)
7	*BRCA2*	c.7806-2A > G	p.?	Bil MBC: IDC, luminal B (62, 66)	M (50)	–
8	*BRCA2*	c.7806-2A > G	p.?	IDC, luminal B (55)	–	FBC (48); 2 PrC (61,64); 2 PaC (74,77)
9	*BRCA2*	c.7806-2A > G	p.?	IDC, luminal A (65)	–	–
10	*BRCA2*	c.7806-2A > G	p.?	Masive DCIS with microinvasion (74)	–	–
11	*BRCA2*	c.3975_3978dupTGCT	p.Ala1327Cysfs*	Mixed IDC and ILC (50)	–	OC (40); FBC (59)
12	*BRCA2*	c.3975_3978dupTGCT	p.Ala1327Cysfs*	IDC, luminal B (68)	–	FBC (30); PrC (72)
13	*BRCA2*	c.3975_3978dupTGCT	p.Ala1327Cysfs*	IDC (63)	–	2 FBC (53,59)
14	*BRCA2*	c.3975_3978dupTGCT	p.Ala1327Cysfs*	IDC, luminal B HER2 + (50)	–	–
15	*BRCA2*	c.3975_3978dupTGCT	p.Ala1327Cysfs*	Masive DCIS with microinvasion (80)	–	3 FBC (41,54,65)
16	*BRCA2*	c.2808_2811delACAA	p.Ala938Profs*	IDC, luminal A (60)	RC(52)	5 FBC (40,40,?)
17	*BRCA2*	c.5560_5561delGT	p.Val1854Phefs*	IDC, luminal B (64)	PrC (51)	OC (62)
18	*BRCA2*	c.6445_6446delAT	p.Ile2149fs*	Bil MBC: IDC, luminal A (62) and IDC, luminal B (64)	–	FBC (49)
19	*BRCA2*	c.6491_6494delAGTT	p.Gln2164Argfs*	IDC luminal B (68)	–	5 FBC (37,43,50,52,62)
20	*BRCA2*	e2-27del	p.?	Encapsulated papillary with invasion (59)	–	FBC (53), PrC (65); PaC (57)

*IDC* invasive ductal carcinoma, *DCIS* ductal carcinoma in situ, *ILC* invasive lobular carcinoma, *Bil* bilateral, *M* melanoma, *RC* renal cell cancer, *PrC* prostate cancer, *PaC* pancreatic cancer, *OC* ovarian cancer.

### Carriers vs. non-carriers

MBC patients who were carries of P/LPVs in *BRCA1* or *BRCA2* were more likely to report a family history of other HBOC-related cancers in first- and/or second-degree relatives (70% vs. 29.5%, *p* = 0.001). While none of the non-carriers developed contralateral breast cancer, both men with bilateral breast cancer in our cohort were carriers of deleterious variants in *BRCA2* (*p* = 0.059). In both unrelated families (2/80) with multiple MBC cases ,deleterious variants in *BRCA2* were identified (*p* = 0.054). Excluding non-melanoma skin cancer and contralateral breast cancer, carriers of deleterious variants in *BRCA1* or *BRCA2* did not have a statistically significant difference in personal history of other cancers (20% vs. 24.6%, *p* = 0.77) or mean age at diagnosis of breast cancer (60.4 vs. 61.2 years, *p* = 0.81) compared to non-carriers.

### Genotype–phenotype correlation

Two out of eight (25%) of identified P/LPVs in *BRCA1* and *BRCA2* were recurrent. The two recurrent ones were *BRCA2*:c.7806-2A > G p.? and *BRCA2*:c.3975_3978dupTGCT p.(Ala1327Cysfs*4), detected in 9 and 5 MBC cases, respectively (Table 2). The first one represented 47.4% (9/19) and the second one 26.3% (5/19) of all P/LPVs in *BRCA2* detected in our MBC cohort.

Since the beginning of genetic counseling and testing at our Institute in 1999, 67 different P/LPVs in *BRCA2* have been detected in 203 unrelated families: 24 variants were recurrent and 43 were detected only once. The two most common recurrent variants were *BRCA2*:c.7806-2A > G p.? and *BRCA2*:c.3975_3978dupTGCT p.(Ala1327Cysfs*4), which were present in 22.2% and 15.8% of the *BRCA2*-positive families, respectively. MBC cases were detected in 10.8% (22/203) of non-related *BRCA2* families. In families with *BRCA2*:c.7806-2A > G, the incidence of MBC cases was higher compared to families with other P/LPVs in *BRCA2*; however, the difference did not reach statistical significance (17.8% vs. 8.9%, *p* = 0.105). Furthermore, *BRCA2*:c.7806-2A > G was detected in both families with multiple cases of MBC in first- and/or second-degree relatives.

MBC patients, who were carriers of *BRCA2*:c.7806-2A > G, did not experience higher incidence of other cancers, excluding non-melanoma skin cancer and contralateral breast cancer (22.2% vs. 23.6%, *p* = 1.0). Also, mean age at diagnosis of breast cancer did not differ between carriers of *BRCA2*:c.7806-2A > G and others (59.6 vs. 61.2 years, *p* = 0.72). Two MBC patients had bilateral breast cancer and both were carriers of P/LPVs: one of them *BRCA2*:c.7806-2A > G and the other *BRCA2*:c.6445_6446delAT (Table 2).

From our database, we also identified all 177 female carriers of P/LPVs in *BRCA2* who developed breast cancer. The most common P/LPV in these women was the recurrent *BRCA2*:c.7806-2A > G detected in 26% (46/177) of these women, followed by the *BRCA2*:c.3975_3978dupTGCT detected in 14.1% (25/177). The splice-site variant represented a significantly higher proportion of all *BRCA2* P/LPVs detected in MBC carriers compared to female breast cancer (FBC) carriers (47.4% vs. 26%, *p* = 0.049).

## Discussion

In our study, we aimed to analyze the prevalence of P/LPVs in *BRCA1* and *BRCA2* genes in the largest cohort of Slovenian MBC patients to date. Our primary objective was to re-evaluate the high detection rate of P/LPVs in *BRCA1* and *BRCA2* as previously reported [9]. We hypothesized the detection rate could be even higher, as more accurate genetic screening methods including next-generation sequencing have been implemented in clinical practice since then. Here, we report a high detection rate (24.7%) of P/LPVs in *BRCA2* (23.5%) and *BRCA1* (1.2%) in the largest cohort of Slovenian MBC patients (*n* = 81). Our cohort is very similar to other published cohorts regarding the age and stage at diagnosis [21–24], histology, and intrinsic subtypes of breast cancer [2, 23, 25–27]. As we predicted, the detection rate is higher than the first reported detection rate of 16% (4/16) in a small cohort of Slovenian MBC patients in 2008 [9]. Then, only limited genetic screening was performed for highly recurrent P/LPVs in *BRCA1* and *BRCA2* detected in Slovenian HBOC families [11] and extended genetic analysis was limited only to those with a substantial family history of breast and/or ovarian cancer [9].

Recently published detection rates of P/LPVs in *BRCA1* and/or *BRCA2* in unselected MBC patients range between 7.8% and 22% in smaller cohorts [28–32]. In two of the largest recently published cohorts, consisting of 382 MBC patients in the collaborative Italian Multicenter Study and 708 mixed population MBC patients tested by Ambry Genetics, California, reported detection rates were 13% and 9%, respectively [33, 34]. Not surprisingly, most deleterious variants were attributed to *BRCA2* and only 1% or less to *BRCA1*, as also seen in our cohort [28, 30–34].

Reported family history of other HBOC-related cancers in 80 unrelated families with MBC cases in our cohort was similar to others (38.7% in our cohort vs. 32% to 39% reported in other unselected MBC cohorts) [30, 32, 33]. We observed a particularly high (43.7%) detection rate in those with a family history of other HBOC-related cancer in first- and/or second-degree relatives. Observed detection rates in our study are in line with high detection rates observed in MBC patients from populations with founder effects [35–37].

Interestingly, almost one third of MBC patients, who were carriers of P/LPVs in *BRCA1* or *BRCA2*, had no family history of other HBOC-related cancers in their first- and/or second-degree relatives. The detection rate in these was high (12.2%). However, these results are similar to those published by other groups who reported between 3.6% and 13% detection rates in MBC patients without any family history of breast cancer [23, 32, 37, 38]. Our findings confirm that genetic testing should be offered to all MBC patients regardless of their family history of other cancers.

In our cohort of MBC patients, the splice-site c.7806-2A > G in *BRCA2* was the most common deleterious variant detected and represented almost half of all variants in *BRCA2* gene. Our group previously reported this variant as a founder Slovenian mutation [10, 11]. It is the most common pathogenic variant in the *BRCA2* gene, detected in Slovenian HBOC families [18], most commonly detected in the central Osrednjeslovenska and eastern Savinjska region of Slovenia [39]. Different published reports on functional analysis of the *BRCA2*:c.7806-2A > G variant showed at least three abnormal transcripts: skipping of entire exon 17, out-of-frame skipping of 20 nucleotides at 5′-end exon 17 and in-frame skipping of 69 nucleotides at 5′-end exon 17 (13 out of 23 amino acids lost being strictly conserved) [14, 40, 41].

This variant was also reported as recurrent in the Italian region Friuli-Venezia-Giulia (FVG) [12–14], which neighbors the western part of Slovenia. Their analysis in 13 Italian carrier families from the FVG region demonstrated that all carriers shared a common haplotype and a common ancestor estimated around 94 generations or 2350 years ago [12]. It is not clear if the haplotype from Italian and Slovenian families is the same as such studies have never been performed [12].

Currently, there is no evidence that would clearly indicate genotype–phenotype correlation between the location of a particular variant within the *BRCA2* gene and predisposition to MBC [42] and no MBC-specific cluster region has been identified [43, 44]. However, it has been reported that some P/LPVs in *BRCA2* were more frequent in MBC cases compared to FBC [36, 37], that male carriers of a specific variant had significantly higher lifetime risks of breast cancer compared to other variants in *BRCA2* [45] and that large genomic rearrangement in the *BRCA2* gene tends to be more frequent in families with MBC cases [46–48].

Possible association between the *BRCA2*:c.7806-2A > G variant and predisposition to MBC was first suggested by the group of researchers from Aviano. Cini et al. found that 39% (7/18) of *BRCA2*:c.7806-2A > G families had MBC cases compared to 16% (13/80) of families with other *BRCA2* P/LPVs (*p* = 0.049) [12]. They also observed a family with this variant and three cases of MBC [12]. To explore possible genotype–phenotype correlation between the splice-site variant and predisposition to MBC in our cohort, we performed additional analysis of all unrelated *BRCA2* families documented at our Institute. This variant was the most frequent among all deleterious variants in *BRCA2* detected in MBC and FBC patients from these families; however, the frequency of the *BRCA2*:c.7806-2A > G variant was significantly higher among MBC compared to FBC carriers (*p* = 0.049). Also, MBC cases were reported twice as often in the *BRCA2*:c.7806-2A > G-positive families compared to families with other P/LPV in BRCA2 identified, but the difference did not reach statistical significance. However, both MBC patients in our cohort with family history of MBC were carriers of this splice-site variant, as was one out of two MBC patients with bilateral breast cancer. As reported by the group from Aviano [12], our analysis also suggest possible genotype–phenotype correlation between the *BRCA2*:c.7806-2A > G variant and predisposition to male breast cancer compared to other deleterious *BRCA2* variants identified at our Institute. However, both studies are small and further research on larger cohorts will be needed. Breast and prostate screening for male carriers of *BRCA1/2* P/LPVs has been offered at our institution for more than 10 years. If carriers of *BRCA2*:c.7806-2A > G variant are in fact at greater risk for developing MBC than other *BRCA2* carriers, their screening recommendations could be modified in view of that risk.

There are several limitations to our study. Although our cohort represents the largest Slovenian set of MBC patients to date, the absolute number of included cases is still small. However, this is a rare disease and Slovenian population is less than 2.1 million people. As this is a retrospective analysis, our cohort is very heterogenous in view of genetic tests performed. We did not identify any P/LPV in *BRCA1* and *BRCA2* in 13 MBC cases who were re-tested using NGS after previously testing negative using other genetic testing methods. Still, some P/LPV could have been missed since in 14 negative MBC cases only limited genetic screening for the six highly recurrent P/LPV in *BRCA1* and *BRCA2* was performed [49]. Since the limited genetic screening in 14 negative MBC cases covered BRCA2:c.7806-2A > G variant but did not include *BRCA2*:c.3975_3978dupTGCT, which was the second most common recurrent variant identified in our cohort, this might led to an underestimated proportion of this variant compared to the c.7806-2A > G among MBC patients in our cohort. Also, variants in other non-*BRCA1*/*BRCA2* genes have been linked to MBC or observed in MBC patients [34, 50], but are not reported in our study. Finally, our study was conducted in a small cohort of patients from the same clinic serving an ethnically homogenous population. Bias due to population stratification could therefore have an important effect on the results of statistical testing.

In conclusion, we observed a high mutation detection rate in this largest cohort of Slovenian MBC patients to date. As predicted, detection rate was even higher than previously reported in a much smaller cohort [9]. We conclude the observed high detection rate may be due to the prevalent Slovenian founder variant *BRCA2*:c.7806-2A > G. As previously suggested by the group of researchers from Aviano [12], our results also indicate a possible association between this splice-site *BRCA2* variant and higher risk of breast cancer in males compared to other identified P/LPVs in *BRCA2* gene. Further research on larger cohorts is needed to explore this. Once genotype–phenotype correlations are better defined, personalized risk assessment and follow-up could be offered to carriers of genetic cancer predispositions.

## Data Availability

The dataset generated and analyzed during the current study is not publicly available due to information that could compromise research participants’ privacy, but is available from the corresponding author AB on reasonable request.

## References

[1] Ottini L (2014). Male breast cancer: A rare disease that might uncover underlying pathways of breast cancer. Nat Rev Cancer.

[2] Giordano SH (2018). Breast cancer in men. N Engl J Med.

[3] Gucalp A, Traina TA, Eisner JR, Parker JS, Selitsky SR, Park BH, Elias AD, Baskin-Bey ES, Cardoso F (2019). Male breast cancer: a disease distinct from female breast cancer. Breast Cancer Res Treat.

[4] Cancer in Slovenia 2017. Institute of Oncology Ljubljana, Epidemiology and Cancer Registry, Slovenian Cancer Registry, 2020. https://www.onko-i.si/fileadmin/onko/datoteke/dokumenti/RRS/lp2017.pdf. Accessed 23 October 2020

[5] Leon-Ferre RA, Giridhar KV, Hieken TJ, Mutter RW, Couch FJ, Jimenez RE, Hawse JR, Boughey JC, Ruddy KJ (2018). A contemporary review of male breast cancer: current evidence and unanswered questions. Cancer Metastasis Rev.

[6] Abdelwahab Yousef AJ (2017). Male Breast Cancer: Epidemiology and Risk Factors. Semin Oncol.

[7] NCCN Guidelines Genetic/Familial High-risk Assessment: Breast, Ovarian, and Pancreatic. V1.2021. https://www.nccn.org/professionals/physician_gls/pdf/genetics_bop.pdf. Accessed 9 October 2020

[8] Hassett MJ, Somerfield MR, Baker ER, Cardoso F, Kansal KJ, Kwait DC, Plichta JK, Ricker C, Roshal A, Ruddy KJ, Safer JD, Van Poznak C, Yung RL, Giordano SH (2020). Management of Male Breast Cancer: ASCO Guideline. J Clin Oncol.

[9] Besic N, Cernivc B, De Grève J, Lokar K, Krajc M, Novakovic S, Zgajnar J, Teugels E (2008). BRCA2 gene mutations in Slovenian male breast cancer patients. Genet Test.

[10] Krajc M, De Grève J, Goelen G, Teugels E (2002). BRCA2 founder mutation in Slovenian breast cancer families. Eur J Hum Genet.

[11] Krajc M, Teugels E, Zgajnar J, Goelen G, Besic N, Novakovic S, Hocevar M, De Grève J (2008). Five recurrent BRCA1/2 mutations are responsible for cancer predisposition in the majority of Slovenian breast cancer families. BMC Med Genet.

[12] Cini G, Mezzavilla M, Della Puppa L, Cupelli E, Fornasin A, D’Elia AV, Dolcetti R, Damante G, Bertok S, Miolo G, Maestro R, de Paoli P, Amoroso A, Viel A (2016). Tracking of the origin of recurrent mutations of the BRCA1 and BRCA2 genes in the North-East of Italy and improved mutation analysis strategy. BMC Med Genet.

[13] Miolo GM, Della Puppa L, Santarosa M, De Giacomi C, Veronesi A, Bidoli E, Tibiletti MG, Viel A, Dolcetti R (2006). Phenotypic features and genetic characterization of male breast cancer families: Identification of two recurrent BRCA2 mutations in north-east of Italy. BMC Cancer.

[14] Santarosa M, Dolcetti R, Magri MD, Crivellari D, Tibiletti MG, Gallo A, Tumolo S, Della PL, Furlan D, Boiocchi M, Viel A (1999). BRCA1 and BRCA2 genes: Role in hereditary breast and ovarian cancer in Italy. Int J Cancer.

[15] Goldhirsch A, Winer EP, Coates AS, Gelber RD, Piccart-Gebhart M, Thürlimann B, Senn HJ, Albain KS, André F, Bergh J, Bonnefoi H, Bretel-Morales D, Burstein H, Cardoso F, Castiglione-Gertsch M, Coates AS, Colleoni M, Costa A, Curigliano G, Davidson NE, Di LA, Ejlertsen B, Forbes JF, Gelber RD, Gnant M, Goldhirsch A, Goodwin P, Goss PE, Harris JR, Hayes DF, Hudis CA, Ingle JN, Jassem J, Jiang Z, Karlsson P, Loibl S, Morrow M, Namer M, Osborne CK, Partridge AH, Penault-Llorca F, Perou CM, Piccart-Gebhart MJ, Pritchard KI, Rutgers EJT, Sedlmayer F, Semiglazov V, Shao ZM, Smith I, Thürlimann B, Toi M, Tutt A, Untch M, Viale G, Watanabe T, Wilcken N, Winer EP, Wood WC (2013). Personalizing the treatment of women with early breast cancer: Highlights of the st gallen international expert consensus on the primary therapy of early breast Cancer 2013. Ann Oncol.

[16] Krivokuca A, Dragos VS, Stamatovic L, Blatnik A, Boljevic I, Stegel V, Rakobradovic J, Skerl P, Jovandic S, Krajc M, Magic MB, Novakovic S (2018). Novel BRCA1 splice-site mutation in ovarian cancer patients of Slavic origin. Fam Cancer.

[17] Bergant G, Maver A, Lovrecic L, Cuturilo G, Hodzic A, Peterlin B (2018). Comprehensive use of extended exome analysis improves diagnostic yield in rare disease: A retrospective survey in 1,059 cases. Genet Med.

[18] Stegel V, Krajc M, Zgajnar J, Teugels E, De Grève J, Hocevar M, Novakovic S (2011). The occurrence of germline BRCA1 and BRCA2 sequence alterations in Slovenian population. BMC Med Genet.

[19] Novakovic S, Milatovíc M, Cerkovnik P, Stegel V, Krajc M, Hocevar M, Zgajnar J, Vakselj A (2012). Novel BRCA1 and BRCA2 pathogenic mutations in Slovene hereditary breast and ovarian cancer families. Int J Oncol.

[20] Klancar G, Blatnik A, Dragos VS, Vogric V, Stegel V, Blatnik O, Drev P, Gazic B, Krajc M, Novakovic S (2020). A novel germline MLH1 in-frame deletion in a Slovenian lynch syndrome family associated with uncommon isolated PMS2 loss in tumor tissue. Genes (Basel).

[21] Yadav S, Karam D, Bin Riaz I, Xie H, Durani U, Duma N, Giridhar KV, Hieken TJ, Boughey JC, Mutter RW, Hawse JR, Jimenez RE, Couch FJ, Leon-Ferre RA, Ruddy KJ (2020). Male breast cancer in the United States: Treatment patterns and prognostic factors in the 21st century. Cancer.

[22] Sarmiento S, McColl M, Musavi L, Gani F, Canner JK, Jacobs L, Fu F, Siotos C, Habibi M (2020). Male breast cancer: a closer look at patient and tumor characteristics and factors that affect survival using the National Cancer Database. Breast Cancer Res Treat.

[23] Deb S, Lakhani SR, Ottini L, Fox SB (2016). The cancer genetics and pathology of male breast cancer. Histopathology.

[24] Mangone L, Ferrari F, Mancuso P, Carrozzi G, Michiara M, Falcini F, Piffer S, Filiberti RA, Caldarella A, Vitale F, Tumino R, Brustolin A, Tagliabue G, Giorgi Rossi P, Ottini L (2020). Epidemiology and biological characteristics of male breast cancer in Italy. Breast Cancer.

[25] Silvestri V, Barrowdale D, Mulligan AM, Neuhausen SL, Fox S, Karlan BY, Mitchell G, James P, Thull DL, Zorn KK, Carter NJ, Nathanson KL, Domchek SM, Rebbeck TR, Ramus SJ, Nussbaum RL, Olopade OI, Rantala J, Yoon SY, Caligo MA, Spugnesi L, Bojesen A, Pedersen IS, Thomassen M, Jensen UB, Toland AE, Senter L, Andrulis IL, Glendon G, Hulick PJ, Imyanitov EN, Greene MH, Mai PL, Singer CF, Rappaport-Fuerhauser C, Kramer G, Vijai J, Offit K, Robson M, Lincoln A, Jacobs L, Machackova E, Foretova L, Navratilova M, Vasickova P, Couch FJ, Hallberg E, Ruddy KJ, Sharma P, Kim SW, Teixeira MR, Pinto P, Montagna M, Matricardi L, Arason A, Johannsson OT, Barkardottir RB, Jakubowska A, Lubinski J, Izquierdo A, Pujana MA, Balmaña J, Diez O, Ivady G, Papp J, Olah E, Kwong A, Nevanlinna H, Aittomäki K, Perez Segura P, Caldes T, Van Maerken T, Poppe B, Claes KBM, Isaacs C, Elan C, Lasset C, Stoppa-Lyonnet D, Barjhoux L, Belotti M, Meindl A, Gehrig A, Sutter C, Engel C, Niederacher D, Steinemann D, Hahnen E, Kast K, Arnold N, Varon-Mateeva R, Wand D, Godwin AK, Evans DG, Frost D, Perkins J, Adlard J, Izatt L, Platte R, Eeles R, Ellis S, Hamann U, Garber J, Fostira F, Fountzilas G, Pasini B, Giannini G, Rizzolo P, Russo A, Cortesi L, Papi L, Varesco L, Palli D, Zanna I, Savarese A, Radice P, Manoukian S, Peissel B, Barile M, Bonanni B, Viel A, Pensotti V, Tommasi S, Peterlongo P, Weitzel JN, Osorio A, Benitez J, McGuffog L, Healey S, Gerdes AM, Ejlertsen B, Hansen TVO, Steele L, Ding YC, Tung N, Janavicius R, Goldgar DE, Buys SS, Daly MB, Bane A, Terry MB, John EM, Southey M, Easton DF, Chenevix-Trench G, Antoniou AC, Ottini L (2016). Male breast cancer in BRCA1 and BRCA2 mutation carriers: Pathology data from the Consortium of Investigators of Modifiers of BRCA1/2. Breast Cancer Res.

[26] Jylling AMB, Jensen V, Lelkaitis G, Christiansen P, Nielsen SS, Lautrup MD (2020). Male breast cancer: clinicopathological characterization of a National Danish cohort 1980–2009. Breast Cancer.

[27] Deb S, Jene N, Investigators K, Fox SB (2012). Genotypic and phenotypic analysis of familial male breast cancer shows under representation of the HER2 and basal subtypes in BRCA-associated carcinomas. BMC Cancer.

[28] Fostira F, Saloustros E, Apostolou P, Vagena A, Kalfakakou D, Mauri D, Tryfonopoulos D, Georgoulias V, Yannoukakos D, Fountzilas G, Konstantopoulou I (2018). Germline deleterious mutations in genes other than BRCA2 are infrequent in male breast cancer. Breast Cancer Res Treat.

[29] Rashid MU, Muhammad N, Naeemi H, Khan FA, Hassan M, Faisal S, Gull S, Amin A, Loya A, Hamann U (2019). Spectrum and prevalence of BRCA1/2 germline mutations in Pakistani breast cancer patients: Results from a large comprehensive study. Hered Cancer Clin Pract.

[30] Scarpitta R, Zanna I, Aretini P, Gambino G, Scatena C, Mei B, Ghilli M, Rossetti E, Roncella M, Congregati C, Bonci F, Naccarato AG, Palli D, Caligo MA (2019). Germline investigation in male breast cancer of DNA repair genes by next-generation sequencing. Breast Cancer Res Treat.

[31] Schayek H, Korach H, Laitman Y, Bernstein-Molho R, Friedman E (2018). Mutational analysis of candidate genes in Israeli male breast cancer cases. Breast Cancer Res Treat.

[32] Ding YC, Steele L, Kuan CJ, Greilac S, Neuhausen SL (2011). Mutations in BRCA2 and PALB2 in male breast cancer cases from the United States. Breast Cancer Res Treat.

[33] Ottini L, Silvestri V, Rizzolo P, Falchetti M, Zanna I, Saieva C, Masala G, Bianchi S, Manoukian S, Barile M, Peterlongo P, Varesco L, Tommasi S, Russo A, Giannini G, Cortesi L, Viel A, Montagna M, Radice P, Palli D (2012). Clinical and pathologic characteristics of BRCA-positive and BRCA-negative male breast cancer patients: Results from a collaborative multicenter study in Italy. Breast Cancer Res Treat.

[34] Pritzlaff M, Summerour P, McFarland R, Li S, Reineke P, Dolinsky JS, Goldgar DE, Shimelis H, Couch FJ, Chao EC, LaDuca H (2017). Male breast cancer in a multi-gene panel testing cohort: insights and unexpected results. Breast Cancer Res Treat.

[35] André S, Pereira T, Silva F, Machado P, Vaz F, Aparício M, Silva GL, Pinto AE (2019). Male breast cancer: Specific biological characteristics and survival in a Portuguese Cohort. Mol Clin Oncol.

[36] Thorlacius S, Sigurdsson S, Bjarnadottir H, Olafsdottir G, Jonasson JG, Tryggvadottir L, Tulinius H, Eyfjörd JE (1997). Study of a single BRCA2 mutation with high carrier frequency in a small population. Am J Hum Genet.

[37] Syrjäkoski K, Kuukasjärvi T, Waltering K, Haraldsson K, Auvinen A, Borg Å, Kainu T, Kallioniemi OP, Koivisto PA (2004). BRCA2 mutations in 154 Finnish male breast cancer patients. Neoplasia.

[38] Frank TS, Deffenbaugh AM, Reid JE, Hulick M, Ward BE, Lingenfelter B, Gumpper KL, Scholl T, Tavtigian SV, Pruss DR, Critchfield GC (2002). Clinical Characteristics of Individuals With Germline Mutations in BRCA1 and BRCA2: Analysis of 10,000 Individuals. J Clin Oncol.

[39] Krajc M, Zadnik V, Novakovic S, Stegel V, Teugels E, Besic N, Hocevar M, Vakselj A, De Grève J, Zgajnar J (2014). Geographical distribution of Slovenian BRCA1/2 families according to family origin: Implications for genetic screening. Clin Genet.

[40] Gelli E, Colombo M, Pinto AM, De Vecchi G, Foglia C, Amitrano S, Morbidoni V, Imperatore V, Manoukian S, Baldassarri M, Lo Rizzo C, Catania L, Frullanti E, Tagliafico E, Cortesi L, Spaggiari F, Mencarelli MA, Trevisson E, Radice P, Renieri A, Ariani F (2019). Usefulness and limitations of comprehensive characterization of mRNA splicing profiles in the definition of the clinical relevance of BRCA1/2 variants of uncertain significance. Cancers (Basel).

[41] Fraile-Bethencourt E, Díez-Gómez B, Velásquez-Zapata V, Acedo A, Sanz DJ, Velasco EA (2017). Functional classification of DNA variants by hybrid minigenes: Identification of 30 spliceogenic variants of BRCA2 exons 17 and 18. PLoS Genet.

[42] Rizzolo P, Silvestri V, Tommasi S, Pinto R, Danza K, Falchetti M, Gulino M, Frati P, Ottini L (2013). Male breast cancer: Genetics, epigenetics, and ethical aspects. Ann Oncol.

[43] Evans DGR, Bulman M, Young K, Howard E, Bayliss S, Wallace A, Lalloo F (2008). BRCA1/2 mutation analysis in male breast cancer families from North West England. Fam Cancer.

[44] Basham VM, Lipscombe JM, Ward JM, Gayther SA, Ponder BAJ, Easton DF, Pharoah PDP (2002). BRCA1 and BRCA2 mutations in a population-based study of male breast cancer. Breast Cancer Res.

[45] Lubinski J, Phelan CM, Ghadirian P, Lynch HT, Garber J, Weber B, Tung N, Horsman D, Isaacs C, Monteiro ANA, Sun P, Narod SA (2004). Cancer variation associated with the position of the mutation in the BRCA2 gene. Fam Cancer.

[46] Woodward AM, Davis TA, Silva AG, Kirk JA, Leary JA (2005). Large genomic rearrangements of both BRCA2 and BRCA1 are a feature of the inherited breast/ovarian cancer phenotype in selected families. J Med Genet.

[47] Tournier I, Brigitte BDP, Sobol H, Stoppa-Lyonnet D, Lidereau R, Barrois M, Mazoyer S, Coulet F, Hardouin A, Chompret A, Lortholary A, Chappuis P, Bourdon V, Bonadona V, Maugard C, Gilbert B, Nogues C, Frébourg T, Tosi M (2004). Significant contribution of germline BRCA2 rearrangements in male breast cancer families. Cancer Res.

[48] Hansen TVO, Jønson L, Albrechtsen A, Andersen MK, Ejlertsen B, Nielsen FC (2009). Large BRCA1 and BRCA2 genomic rearrangements in Danish high risk breast-ovarian cancer families. Breast Cancer Res Treat.

[49] Rizzolo P, Silvestri V, Ottini L (2017). Retesting BRCA1/BRCA2 mutation negative male breast cancer patients using next generation sequencing technologies. Breast Cancer Res Treat.

[50] Rizzolo P, Zelli V, Silvestri V, Valentini V, Zanna I, Bianchi S, Masala G, Spinelli AM, Tibiletti MG, Russo A, Varesco L, Giannini G, Capalbo C, Calistri D, Cortesi L, Viel A, Bonanni B, Azzollini J, Manoukian S, Montagna M, Peterlongo P, Radice P, Palli D, Ottini L (2019). Insight into genetic susceptibility to male breast cancer by multigene panel testing: Results from a multicenter study in Italy. Int J Cancer.

